# Negative emotional symptoms and e-Health literacy among Chinese college students: a latent profile analysis

**DOI:** 10.3389/fpsyg.2026.1760468

**Published:** 2026-03-04

**Authors:** Di Dai, Qingping Zhou, Yusupujiang Tuersun, Yuying Xie, Yao Yu, Siyuan Liu, Chenxi Wang, Zhenning Liang, Yi Qian

**Affiliations:** 1 School of Health Management, Southern Medical University, Guangzhou, China; 2 Shenzhen Longhua District Maternity and Child Health Hospital, Shenzhen, China; 3 School of Public Health, Southern Medical University, Guangzhou, China; 4 Department of Education, The Seventh Affiliated Hospital, Sun Yat-sen University, Shenzhen, China

**Keywords:** Chinese college students, cross-sectional study, e-Health literacy, latent profile analysis, negative emotional symptoms

## Abstract

**Background:**

Negative Emotional symptoms such as depression and anxiety do not exist independently, often co-occurring in the same individual, and heterogeneity exists between individuals suffering from depression and anxiety; however, prior research has rarely investigated heterogeneity in a person-centered manner and from the perspective of college students. The main purpose of this study was to explore this heterogeneity and its association with e-Health literacy (e-HL) using Latent profile analysis (LPA), a person-centered statistical method.

**Method:**

A total of 7,503 Chinese college students from 10 regions (including Guangdong Province, Shanghai Municipality, and Jiangsu Province) were surveyed using the Generalized Anxiety Disorder Scale (GAD-7) and Patient Health Questionnaire (PHQ-9) to assess anxiety and depressive symptoms. LPA was employed to identify potential profiles of negative emotional symptoms and validate their robustness; binary logistic regression was used to explore differences in demographic characteristics (sex, grade ranking), sociological factors (family residential background, per capita monthly family income), and lifestyle factors (adherence to physical activity, smoking status, alcohol consumption) across profiles; analysis of variance (ANOVA) was applied to compare e-HL levels among different profiles.

**Results:**

The two-class model was identified as the optimal classification of negative emotional symptoms: low/no negative emotional symptoms (61.49%) and high negative emotional symptoms (38.51%). Female college students, those with low per capita monthly family income, lack of regular physical exercise, and alcohol consumption habits were more likely to be categorized into the high negative emotional symptoms group (all *p* < 0.001). E-Health literacy levels were significantly negatively correlated with the severity of negative emotional symptoms (*F* = 212.661, *p* < 0.001), with the low/no negative emotional symptoms group showing higher average e-HL scores (30.11 ± 7.004 vs. 27.80 ± 5.837).

**Limitations:**

Reliance on self-report measures may lead to recall bias and social desirability bias; the cross-sectional design cannot establish causal relationships between variables; digital addiction, a potential confounding factor that may co-occur with negative emotional symptoms and influence e-HL, was not included in the analysis.

**Conclusion:**

This study identified two distinct latent profiles of negative emotional symptoms among Chinese college students and their key predictive factors using LPA. The findings highlight the need for stratified early screening for high-risk groups (females, low-income families, inactive individuals, and drinkers) and the development of targeted interventions. Enhancing e-HL could be a potential pathway to improve mental health outcomes, providing actionable insights for scientific and effective mental health management in colleges and universities.

## 1 Introduction

China’s educational philosophy is deeply influenced by Confucianism, Confucian values emphasize academic achievement as a core life goal and impose expectations of “bringing honor to the family,” leading college students face multiple pressures including academic demands, parental expectations, and peer competition (Chang et al., 2020), which increase their risk of mental health problems. Previous studies have shown that Chinese college students exhibit declining physical fitness, high sub-health prevalence, and suboptimal overall mental health (Ning et al., 2024; Rivas et al., 2020; Xia et al., 2023). Negative emotional symptoms refer to transient, situation-related emotional experiences such as anxiety and depression that are unpleasant, uncomfortable, or distressing, which are usually associated with negative evaluations, unmet needs, or difficulties experienced by the individual (Eisenberg et al., 2007). When negative emotional symptoms are too strong, too long-lasting, or inconsistent with reality, they may negatively impact an individual’s mental health and quality of life. It is common for college students to experience negative emotional symptoms, which can lead to suicide risk and serious impairments in functioning and quality of life (Lei et al., 2024; Galera et al., 2021). Thus, investigating the characteristics and influencing factors of negative emotional symptoms in this population is of great public health significance.

Negative emotional symptoms such as depression and anxiety are strongly correlated and often comorbid (Axelson and Birmaher, 2001), with confirmatory factor analyses supporting their structural association (Snyder et al., 2009). Moreover, significant heterogeneity exists in symptom presentation among individuals with these symptoms (Choi et al., 2020). Unfortunately, most prior studies have adopted a variable-oriented approach, which may overlook symptom-specific differences and key clinical distinctions (Hou and Zhang, 2023). Notably, existing research has two critical gaps: first, few studies have used person-centered methods to explore the latent heterogeneity of negative emotional symptoms in college students; second, the specific mechanism linking distinct negative emotional symptom profiles to e-Health literacy (e-HL) remains under-theorized, with no clear theoretical framework explaining their interaction. Latent profile analysis (LPA) provides a solution to the problem of heterogeneity between depression and anxiety symptoms. LPA is an individual-centered statistical analysis that categorizes different latent subgroups and observes their population characteristics and heterogeneity according to the response patterns of individuals on the exogenous variables (Bajenaru et al., 2022; Berlin et al., 2014). Compared to cluster analysis, LPA categorization is more objective and results are more accurate, and it also clarifies the nature and number of categories (Luo et al., 2024). This method scales participants into profile categories based on similar feedback results. LPA is also able to identify potential profile patterns for combination studies of different symptoms. For example, Wang et al. (2021) examined patterns of depression and anxiety in youth affected by the Lushan earthquake and identified three distinct categories. Dai et al. (2024) investigated depression and anxiety symptoms in 7,422 adolescents from 23 primary and secondary schools from China and also identified three distinct patterns of depression and anxiety; while Wang et al. (2024) analyzed these 7,422 adolescents for patterns of Internet addiction, identifying four categories: routine, risky, low Internet addiction, and Internet addiction. Similarly, Albuquerque et al. (2024) analyzed data from 918 university students from Brazil and identified three different categories associated with mental illness. Nonetheless, there are still significant gaps in research exploring potential profile categorization in college student populations. Identifying the different symptom profiles exhibited by negative emotional symptoms in Chinese college student populations is crucial for developing individualized treatment interventions. Therefore, the present study aimed to examine the heterogeneity of anxiety symptoms and depressive symptoms in negative emotional symptoms among Chinese college students through LPA.

Demographic, sociological, and lifestyle factors are known predictors of anxiety and depression. Females are more prone to negative emotions than males, this difference may be attributed to biological factors, sociocultural norms, and coping styles (Tao et al., 2024; Liu et al., 2019; Zhang X. et al., 2024). In addition, the sociological characteristics of college students and their personal lifestyles were also able to predict their anxiety and depression (Zhou et al., 2024). Zeng et al. (2024) surveyed 1,555 college students from five universities in China and found that better family functioning was associated with fewer psychological symptoms and identified three mental health categories that correlated with psychological symptom severity. Results of a survey from South Africa show that mental health conditions such as anxiety and depression are related to physical activity participation among university students (Johannes et al., 2024); The results of a meta-analysis suggest that students in higher education often suffer from stress-induced physical and mental health problems, which may have a negative impact on their academic performance and lead to a relatively low grade ranking (Wunsch et al., 2021). Therefore, this study included sex, grade ranking, family residential background, per capita monthly family income, adherence to physical activity, smoking status, and alcohol consumption as predictors.

E-Health literacy is defined as the ability to search, screen, understand, evaluate, and utilize online health information, which closely associated with mental health. This study integrates Social Cognitive Theory (SCT) with the eHealth literacy model into a coherent theoretical framework to explain the relationship between negative emotional symptoms and eHealth literacy (Bandura and Health NIOM, 1986). From the SCT perspective, negative emotional symptoms reduce individuals’ self-efficacy via persistent negative cognitions. This impairs motivation and ability to engage in core e-HL competencies, as anxiety conversely, the e-HL Model posits that application, judgmental, and decision-making competencies enable access to evidence-based mental health resources, alleviating negative emotions by addressing underlying stressors. In addition, to further substantiate the LPA classification, this study investigated and analyzed the differences in e-HL among different groups of college students. Previous research has shown that negative emotional symptoms such as anxiety and depression are strongly associated with e-HL levels, and that college students with higher levels of anxiety and depressive symptoms have lower levels of e-HL (Zhao et al., 2023; Castarlenas et al., 2021). This study aimed to validate LPA classification by examining e-HL differences across profiles. Three hypotheses were proposed: (1) Multiple latent profiles of negative emotions can be identified based on anxiety and depressive symptom severity; (2) Female sex, low grade ranking, rural family residential background, low per capita monthly family income, lack of physical activity, smoking, and alcohol consumption are associated with higher negative emotion symptom levels; (3) e-HL is negatively correlated with the severity of negative emotion symptoms.

## 2 Method

### 2.1 Participants

**Figure 1:**
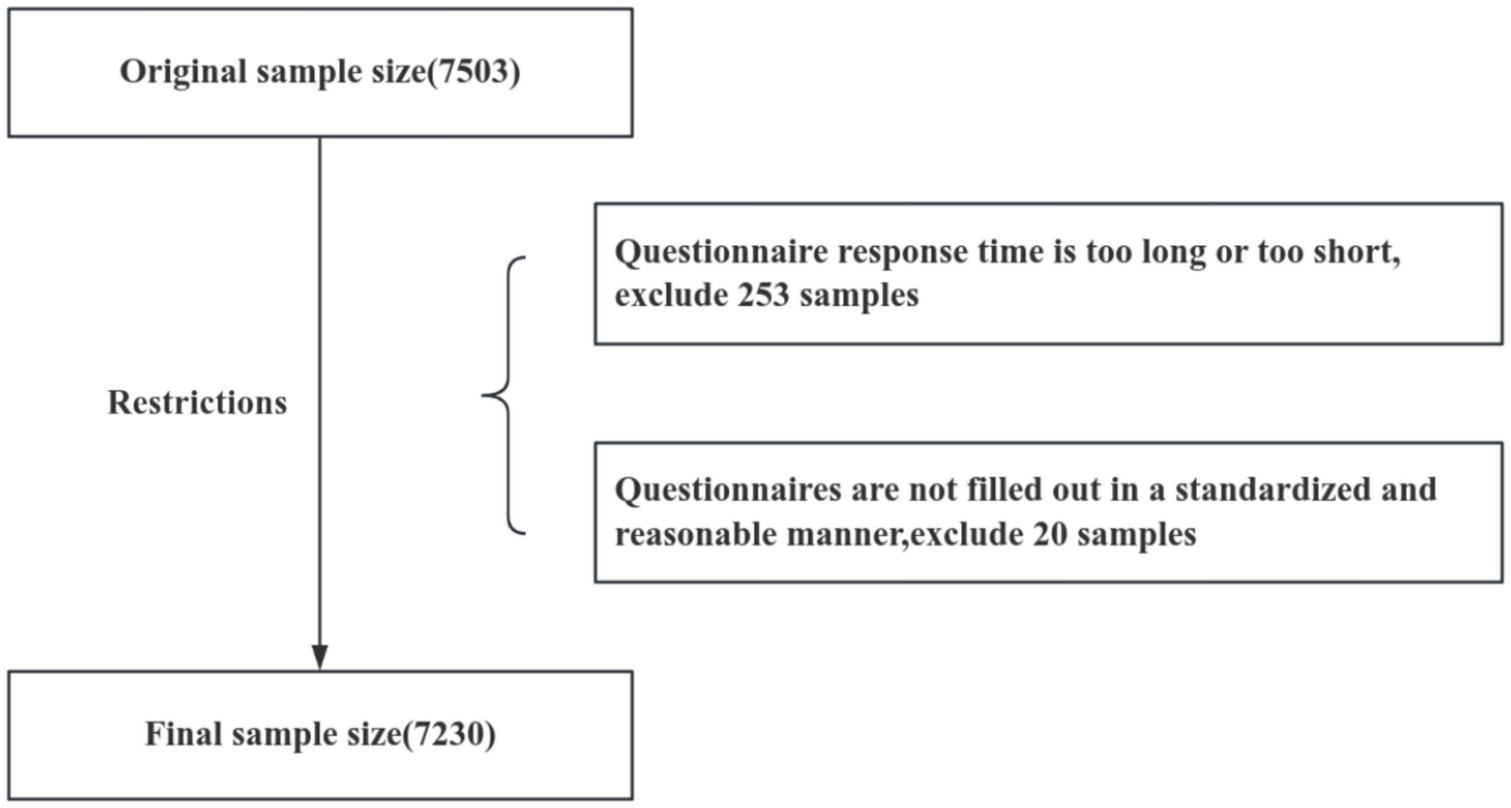
Flowchart of study participant.

From January to February 2023, 7,503 college students (freshmen to seniors) from 10 regions in China were invited to participate via the Questionnaire Star platform. After excluding questionnaires with abnormal response times (253 samples, <5 min or >30 min) and invalid responses (20 samples, including inconsistent answers to validation questions or logical contradictions in key items), 7,230 valid questionnaires were retained (effective recovery rate: 96.3%) (Figure 1).

### 2.2 Procedure

The study was approved by the Ethics Committee of the first author’s university [Southern Medical University Audit (2023) No. 46]. A convenience sampling method was used to distribute an anonymous electronic questionnaire through the Questionnaire Star platform^1^, with the following inclusion criteria: (1) currently enrolled college students; (2) consistent answers to 2 validation questions. Exclusion criteria: (1) self-reported cognitive impairment or inability to complete the questionnaire independently; (2) unwillingness to provide informed consent. Participants completed demographic, sociological, and lifestyle information, as well as the GAD-7, PHQ-9, and e-Health Literacy Scale, which took approximately 15 min.

### 2.3 Measures

#### 2.3.1 Negative emotions

This study synthesized the status of negative emotional symptoms in college students through the profile of anxiety symptoms and depressive symptoms. The GAD-7 was used to investigate anxiety symptoms in study participants (Crocq, 2017). The GAD-7 consists of 7 entries, each scored on a 4-point scale, with scores ranging from 0.0–3.0 for “not at all,” “a few days,” “more than half the time,” and “almost every day”, with scores ranging from 0 to 21. A score of <5.0 indicates no anxiety symptoms, while a score of >5.0 is considered to indicate anxiety symptoms. The choice of the cutoff value >5.0 was based on the study’s focus on early screening of subclinical symptoms in college students, this threshold allows for the identification of mild anxiety symptoms, which is critical for timely intervention in young adults (Moreno et al., 2019). Although the standard cutoff for moderate anxiety is ≥10.0, this study prioritizes early detection of potential mental health risks in the general college population rather than diagnosing clinical cases. Commonly used internationally to screen individuals for anxiety levels, it has good psychometric properties and good reliability in previous studies, with a Cronbach’s alpha coefficient of 0.928 (Huang et al., 2018). The Cronbach’s alpha coefficient for GAD-7 in the sample of this study was 0.944. The PHQ-9 was used to investigate depressive symptoms in study participants. The PHQ-9 consists of 9 entries; scoring is identical to the GAD-7. A score of <5.0 indicates no depressive symptoms, while >5.0 is considered to indicate depressive symptoms. Similarly, the cutoff >5.0 for PHQ-9 was chosen to screen for mild depressive symptoms, aligning with the early intervention goal for college students (Kroenke et al., 2001). The internal consistency estimate of the PHQ-9 has been repeatedly validated, with a Cronbach’s alpha coefficient of 0.859 in previous study samples (Salcioglu et al., 2018). The Cronbach’s alpha coefficient for the PHQ-9 in the present study sample was 0.931.

#### 2.3.2 e-Health literacy

In this study, the e-Health Literacy Scale was used to assess the e-HL level of college students. The e-Health Literacy Scale was developed in 2016 by Norman and Skianer and other scholars based on the concept of e-HL and the Lily model to evaluate an individual’s ability to search, screen, understand, evaluate, and utilize health information (Norman and Skinner, 2006). The scale consists of 8 entries, which are categorized into 3 different online health information and service competencies based on the different connotations of the 8 entries of the e-Health Literacy Scale: application competency (entries 1–5), judgmental competency (entries 6–7), and decision-making competency (entry 8). The scale was based on a five-point Likert scale, ranging from 1 to 5, from “strongly disagree” to “strongly agree,” with 8 entries totaling 8–40 points; the higher the total score, the higher the level of e-HL, and 32 points was the passing standard (Park et al., 2016; Chung and Nahm, 2015). The Cronbach’s alpha coefficient for this study was 0.974. Item-total correlation analysis showed that all items were significantly correlated with the total score (*r* = 0.78–0.89, all *p* < 0.001), indicating no obvious item redundancy. The McDonald’s omega coefficient was 0.968, confirming good internal consistency.

#### 2.3.3 Demographic and sociological characteristics

Demographic characteristics include sex, which is categorized as male and female. Sociological characteristics included grade ranking, family type, per capita monthly family income. Grade ranking was divided into four categories: <25.0, 25.0–50.0%, 50.0–75.0%, and ≥75.0%; respondents were categorized into rural and urban based on the family residential background filled out in the survey; and per capita monthly household income was also categorized into four categories: <2,500 RMB, 2,500–5,000 RMB, 5,000–10,000 RMB, and ≥10,000 RMB.

#### 2.3.4 Personal lifestyle

The three variables of adherence to physical activity, smoking status and alcohol consumption were, respectively, categorized as yes or no according to “exercising more than three times a week for more than 30 minutes each time in the past month” (Organization WH, 2020), “smoking in the past month”, and “drinking alcohol (including liquor, beer, wine, or yellow wine, etc.) in the past month”.

### 2.4 Data analysis

In this study, data analysis was conducted using Mplus version 8.3 and SPSS 23.0^2^. To determine the heterogeneity of negative emotional symptom presentation, we assessed the respondents’ anxiety and depression symptoms using the GAD-7 and PHQ-9, and performed Latent Profile Analysis (LPA). Models containing 1 to 5 latent classes were ordered to identify the most meaningful and parsimonious model. Six fit indices were used to evaluate the models: Akaike Information Criterion (AIC), Bayesian Information Criterion (BIC), sample size-adjusted BIC (aBIC), Lo–Mendell–Rubin likelihood ratio test (LMRT), bootstrap likelihood ratio test (BLRT), and entropy. Generally, the best-fitting model should exhibit lower AIC, BIC, and aBIC values; higher entropy; significant LMRT and BLRT values; and each subgroup should account for at least 5% of the total population. In addition to fit statistics, model selection should also consider previous research and the clinical significance of the latent classes.

Secondly, some variables were used as predictors for the optimal latent profile analysis model. We performed binary logistic regression using the three-step command in Mplus 8.3 to evaluate whether the selected variables as predictors would lead to a higher probability of a college student belonging to one latent class over another. The LPA results served as the dependent variable, while gender, grade ranking, family type, per capita monthly family income, adherence to physical activity, smoking status and alcohol consumption were used as independent variables. Binary logistic regression analysis was conducted, and odds ratios (OR) and their 95% confidence intervals (95% CI) were calculated based on the *β* values and standard errors, and reported in the results section. The OR values reflect the predictive effects of gender, grade ranking, family type, per capita monthly family income, adherence to physical activity, smoking status and alcohol consumption on the negative emotions of different profile classes.

Finally, the BCH (Bias-Corrected Bootstrap with a Huber-White estimator) method was applied to analyze e-HL as an outcome measure of negative emotional states. All statistical analyses were performed using Mplus version 8.3 and SPSS 23.0 software, with a two-sided test *p* < 0.05 considered statistically significant. The design and analysis of this study were not pre-registered.

## 3 Result

### 3.1 Descriptive statistics and correlation analysis

The average GAD-7 score was 3.72 ± 4.00, and the average PHQ-9 score was 4.90 ± 5.05, as detailed in Table 1. Correlation analysis showed that negative emotional symptoms (anxiety and depression) were significantly positively correlated with sex (anxiety: *r* = 0.126, *p* < 0.001; depression: *r* = 0.115, *p* < 0.001) and alcohol consumption (anxiety: *r* = 0.058, *p* < 0.001; depression: *r* = 0.069, *p* < 0.001). Negative emotional symptoms were significantly negatively correlated with family residential background (anxiety: *r* = −0.078, *p* = 0.002; depression: *r* = −0.024, *p* = 0.040), per capita monthly family income (anxiety: *r* = −0.092, *p* < 0.001; depression: *r* = −0.086, *p* < 0.001), adherence to physical activity (anxiety: *r* = −0.119, *p* < 0.001; depression: *r* = −0.146, *p* < 0.001), and e-HL (anxiety: *r* = −0.205, *p* < 0.001; depression: *r* = −0.214, *p* < 0.001). Collinearity analysis showed variance inflation factors (VIF) < 10 for all independent variables, indicating no multicollinearity (Table 2).

**Table 1:** Basic information on each variable for each potential profile (*n* = 7,230).

Variables	Overall *n* = 7,230 (100.00%)	Class 1 *n* = 4,446 (61.49%)	Class 2 *n* = 2,784 (38.51%)	*x*^2^/F	*p*-value
Sex				39.123	<0.001
Male	2,410 (33.33%)	1,604 (36.08%)	806 (28.95%)		
Female	4,820 (66.67%)	2,842 (63.92%)	1978 (71.05%)		
Grade ranking				14.051	0.003
<25.0%	1998 (27.63%)	1,202 (27.04%)	796 (28.59%)		
25.0–50.0%	2,649 (36.65%)	1,672 (37.61%)	977 (35.09%)		
50.0–75.0%	1891 (26.15%)	1,184 (26.63%)	707 (25.40%)		
≥75.0%	692 (9.57%)	388 (8.72%)	304 (10.92%)		
Family residential background				7.631	0.006
Rural	4,510 (62.38%)	2,718 (61.13%)	1792 (64.37%)		
Urban	2,720 (37.62%)	1728 (38.87%)	992 (35.63%)		
Per capita monthly family income				53.149	<0.001
<2500RMB	1,377 (19.05%)	741 (16.67%)	636 (22.84%)		
2,500–5000RMB	3,015 (41.70%)	1867 (41.99%)	1,148 (41.24%)		
5,000–10000RMB	1982 (27.41%)	1,255 (28.23%)	727 (26.11%)		
≥10000RMB	856 (11.84%)	583 (13.11%)	273 (9.81%)		
Adherence to physical activity				145.247	<0.001
No	2,136 (29.54%)	1,086 (24.43%)	1,050 (37.72%)		
Yes	5,094 (70.46%)	3,360 (75.57%)	1734 (62.28%)		
Smoking status				0.368	0.544
No	7,005 (96.89%)	4,312 (96.99%)	2,693 (96.73%)		
Yes	225 (3.11%)	134 (3.01%)	91 (3.27%)		
Alcohol consumption				25.811	<0.001
No	6,055 (83.75%)	3,801 (85.49%)	2,254 (80.96%)		
Yes	1,175 (16.25%)	645 (14.51%)	530 (19.04%)		
GAD-7 (mean ± SD)	3.72 ± 4.00	1.21 ± 1.60	7.72 ± 3.39	12099.150	<0.001
PHQ-9 (mean ± SD)	4.90 ± 5.05	1.81 ± 2.22	9.84 ± 4.32	10781.682	<0.001
e-Health literacy (mean ± SD)	29.22 ± 6.68	30.11 ± 7.00	27.80 ± 5.84	212.661	<0.001

Class 1 = low/no negative emotional symptoms; Class 2 = high negative emotional symptoms.

**Table 2:** Bivariate correlations for all study variables.

Variables	1	2	3	4	5	6	7	8	9	10
1. Sex	1									
2. Grade ranking	−0.089**	1								
3. Family type	−0.090**	−0.030*	1							
4. Per capita monthly family income	−0.087**	−0.056**	0.365**	1						
5. Adherence to physical activity	0.002	−0.088**	0.062**	0.079**	1					
6. Smoking status	−0.189**	0.067**	−0.001	0.005	−0.043**	1				
7. Alcohol consumption	−0.176**	0.028*	0.083**	0.083**	−0.020	0.275**	1			
8. e-Health literacy	−0.044**	−0.078**	0.173**	0.165**	0.341**	−0.029*	−0.005	1		
9. GAD-7	0.126**	−0.002	−0.037**	−0.092**	−0.119**	−0.005	0.058**	−0.205**	1	
10. PHQ-9	0.115**	0.010	−0.024*	−0.086**	−0.146**	−0.004	0.069**	−0.214**	0.843**	1

*p < 0.05; **p < 0.01.

### 3.2 Latent profile analysis

This study began with an initial model of one class and progressively increased the number of model classes to five. We evaluated and tested the fit and differences of the latent profile models using various indices and integrated theoretical rationale, clinical interpretability, and statistical fit to determine the optimal model. The indices for different LPA models are presented in Table 3.

**Table 3:** Fit indices for one-to-five-profile models of latent profile analysis.

Profile	AIC	BIC	aBIC	LMRT	BLRT	Entropy	Latent profile proportion (%)
				*P* value			
1	238187.637	238407.989	238306.3				
2	182727.195	183064.608	182908.897	0.000	0.000	0.965	61.49/38.51
3	155668.424	156122.899	155913.166	0.000	0.000	0.977	56.61/39.27/4.12
4	147844.517	148416.054	148152.299	0.125	0.000	0.942	44.86/21.78/29.83/3.53
5	141916.337	142604.936	142287.158	0.104	0.000	0.951	43.20/21.58/29.28/4.48/1.46

1. AIC = Akaike Information Criterion; BIC=Bayesian Information Criterion; aBIC = sample size-adjusted BIC; LMRT = Lo–Mendell–Rubin likelihood ratio test; BLRT = bootstrap likelihood ratio test. 2. Lower AIC/BIC/aBIC and higher entropy indicate better model fit; significant LMRT/BLRT (*p* < 0.05) indicates the current model is superior to the model with one fewer class. 3. The two-class model was selected based on integrated evaluation of: (a) significant LMRT/BLRT; (b) excellent entropy (>0.90); (c) avoidance of overfitting and non-interpretable small classes.

Considering the BLRT, the *p*-values for each class model were significant at the *α* = 0.05 level, and the entropy values for all 2–5 class models were acceptable (all above 0.90). As the number of latent classes increased, the AIC, BIC, and aBIC values continued to decrease. Therefore, these fit indices might not be very helpful in selecting the appropriate model.

However, the *p*-values of the LMR were significant (*p* < 0.01) in the two-class and three-class models, but not significant in the four-class (*p* = 0.125) and five-class models (*p* = 0.104). This indicates that the two-class model is better than the one-class model, and the three-class model is better than the two-class model, but the four-class model is not better than the three-class model, and the five-class model is not better than the four-class model.

The three-class solution included low negative emotional symptoms (56.61%), moderate negative emotional symptoms (39.27%), and high negative emotional symptoms (4.12%). Although the LMRT was significant, the smallest class (high negative emotional symptoms) accounted for only 4.12% of the sample. Clinically, this intermediate group lacked distinct characteristics to guide targeted interventions, as their mental health needs could not be differentiated from the low or high groups. Additionally, the 4.12% small class, while statistically detectable in a large sample (*n* = 7,230), had limited generalizability and practical value for population-based mental health screening.

The two-class solution was supported by strong theoretical and clinical rationale. Theoretically, it aligns with the “subclinical-clinical” dichotomy of mental health in general populations, which is widely used in public health screening. Clinically, this classification clearly distinguishes high-risk groups that require targeted interventions from low-risk groups that need preventive education—an essential distinction for college mental health management. Based on the integrated evaluation of statistical fit, theoretical consistency, and clinical applicability, the two-class model was identified as the optimal solution.”

**Figure 2:**
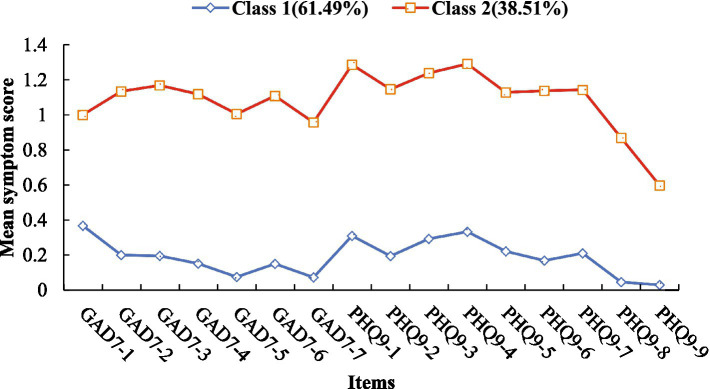
Each item scores of the PHQ-9 and GAD-7 for the two-class model’s profile plot. Class 1 = low/no negative emotional symptoms; Class 2 = high negative emotional symptoms.

The two-class characteristics were low/no negative emotional symptoms (61.49%) and high negative emotional symptoms (38.51%), as shown in Table 3. The average latent class probabilities for each group in the two-class model were all greater than 0.90, as shown in Table 4. The scores for each item of the GAD-7 and PHQ-9 in the two-class model are shown in Figure 2. Analysis of variance showed that there were significant differences in each item of the GAD-7 and PHQ-9 (*p* < 0.001), as shown in Table 5.

**Table 4:** Average latent class probabilities for most likely latent class membership (Row) by latent class (Column).

Average latent class probabilities for most likely latent class membership (Row) by latent class (Column)		
Class	1	2
1	0.991	0.009
2	0.013	0.987

**Table 5:** Mean differences across items of negative emotions in potential profiles.

Items	Class 1	Class 2	*F*
GAD7-1	0.370 (0.494)	1.220 (0.607)	4257.646***
GAD7-2	0.200 (0.406)	1.130 (0.619)	5993.405***
GAD7-3	0.200 (0.403)	1.170 (0.608)	6681.435***
GAD7-4	0.150 (0.360)	1.120 (0.568)	7890.823***
GAD7-5	0.070 (0.267)	1.010 (0.620)	7763.446***
GAD7-6	0.150 (0.366)	1.110 (0.599)	7112.136***
GAD7-7	0.070 (0.269)	0.960 (0.672)	6171.635***
PHQ9-1	0.310 (0.508)	1.290 (0.666)	4956.031***
PHQ9-2	0.190 (0.401)	1.150 (0.568)	6953.063***
PHQ9-3	0.290 (0.531)	1.240 (0.759)	3862.620***
PHQ9-4	0.330 (0.517)	1.290 (0.635)	4910.181***
PHQ9-5	0.220 (0.453)	1.130 (0.702)	4434.485***
PHQ9-6	0.170 (0.407)	1.140 (0.700)	5529.613***
PHQ9-7	0.210 (0.468)	1.140 (0.723)	4445.404***
PHQ9-8	0.050 (0.223)	0.870 (0.716)	5061.600***
PHQ9-9	0.030 (0.177)	0.600 (0.728)	2467.533***

Class 1 = low/no negative emotional symptoms; Class 2 = high negative emotional symptoms. ***p < 0.001.

### 3.3 Predictors of latent profile membership

Binary logistic regression showed that sex, per capita monthly family income, adherence to physical activity, and alcohol consumption were significant predictors of profile membership (Table 6).

**Table 6:** Predictors of latent class groups.

Variables	Class 1 as reference		
	Class 2		
	*B*	SE	OR(95%CI)
Sex	0.370***	0.055	1.448(1.300–1.613)
Grade ranking	−0.006	0.026	0.994(0.944–1.047)
Family residential background	0.009	0.055	1.009(0.906–1.123)
Per capita monthly family income	−0.162***	0.029	0.850(0.803–0.900)
Adherence to physical activity	−0.608***	0.067	0.544(0.490–0.604)
Alcohol consumption	0.444***	0.069	1.558(1.360–1.785)

Class 1 = low/no negative emotional symptoms; Class 2 = high negative emotional symptoms. *p < 0.05; **p < 0.01; ***p < 0.001.

Females were more likely to be in the high negative emotional symptoms group than males (OR = 1.448, *p* < 0.001); Lower per capita monthly family income was associated with higher odds of being in the high negative emotional symptoms group (OR = 0.850, *p* < 0.001); Regular physical activity was a protective factor (OR = 0.544, *p* < 0.001); Alcohol consumption was associated with higher odds of being in the high negative emotional symptoms group (OR = 1.558, *p* < 0.001). Grade ranking and family residential background were not significant predictors (*p* > 0.05).

### 3.4 Differences in e-Health literacy across latent profiles

In this study, e-HL was analyzed as a variable associated with negative emotional symptom characteristics using the BCH approach. As shown in Table 7, there were significant differences in e-HL between the two-class models. The average score of e-HL was higher in the low/no negative emotional symptom group, indicating that the higher the severity of negative emotional symptom, the lower the level of e-HL.

**Table 7:** Parameter estimates of e-Health literacy on the two profiles, M (SE).

Variables	*M* (SE)	Pairwise comparisons (*x*^2^)
E-HL	Class 1 = 30.135 (0.106)	231.124***
	Class 2 = 27.766 (0.112)	

Class 1 = low/no negative emotional symptom; Class 2 = high negative emotional symptom. ***p < 0.001.

## 4 Discussion

To our knowledge, this is one of the first studies to use LPA to identify latent subgroups of negative emotional symptom among Chinese college students, confirming a two-class model (low/no negative emotional symptom and high negative emotional symptom). Sex, per capita monthly family income, adherence to physical activity, and alcohol consumption were identified as key predictors, and e-HL levels differed significantly across profiles. These findings have important implications for college mental health management.

### 4.1 Two-class latent parallel model

Firstly, we adopted a person-centered approach to assess the heterogeneity of negative emotional symptom presentation (Hu et al., 2022). On the one hand, we found that the differences between the classification models were only in the severity of negative emotion symptoms, rather than the type of symptoms. In short, the classification model can only identify based on the severity of symptoms rather than the absence or presence of specific symptoms. This reflects a high similarity in the pattern of symptom severity response for these distress disorders, which can provide references for clinical assessment and/or treatment. Our study also found that within each latent profile category, the severity of individual negative emotions exhibited flat differences, meaning that the severity reflected by depression and anxiety in negative emotions is consistent with each other, and there are no subgroups dominated by depressive symptoms or anxiety symptoms. This result is consistent with previous studies (Dai et al., 2024), suggesting that in practice, for patients with negative emotions, the possibility of comorbidity of anxiety and depression should be considered (Wang et al., 2020).

Additionally, item 1 of the GAD-7 (“Feeling nervous, anxious, or on edge”) scored significantly higher than other anxiety-related items. This may be because academic pressures such as performance requirements, exams, study materials, and time management make students feel tense and uneasy. Academics are an indispensable part of every college student’s life, and without a healthy attitude towards academic goals, students may experience severe stress. While academics can be seen as a positive challenge with the potential to enhance learning abilities and skills, if viewed negatively, this pressure may adversely affect students’ mental health (Beiter et al., 2015); For the PHQ-9, item 1 (“Little interest or pleasure in doing things”) and item 4 (“Feeling tired or having little energy”) scored significantly higher than other depression-related items. This may be because sleep disorders (SD) are prevalent among college students (Nyer et al., 2013). Poor sleep quality and insufficient rest lead to a lack of interest in activities and feelings of tiredness or lack of energy. Sleep disorders (SD) are common somatic symptoms in patients with major depressive disorder (MDD), including excessive or insufficient sleep and restless sleep, and are also one of the DSM-IV diagnostic criteria for depression (Vanwoerden and Stepp, 2022); It is worth noting that item 9 of the PHQ-9 (“Thoughts that you would be better off dead, or of hurting yourself in some way”) scored significantly lower than other items, indicating that suicidal ideation among college students in this study was relatively low, consistent with previous research on suicidal ideation among Chinese college students (Kang et al., 2023; Cong et al., 2021). Suicidal ideation is the most important predictor of suicidal behavior (Liao et al., 2022), and represents the individual’s thoughts and intentions of ending their life (Grasdalsmoen et al., 2020). Notably, the average GAD-7 (3.72 ± 4.00) and PHQ-9 (4.90 ± 5.05) scores in this sample were relatively low (below the clinical cutoff of ≥10), which may reflect the overall mental health status of Chinese college students in non-clinical settings. This finding suggests that our two-profile model is particularly suitable for population-based early screening, as it identifies high-risk individuals within a generally low-severity population, this is more clinically meaningful than a median split, which would arbitrarily divide the population into two groups regardless of clinical relevance. Compared to median splits, LPA identifies latent subgroups based on empirical response patterns, avoiding arbitrary cutoff points and better capturing the true heterogeneity of symptom presentation.

### 4.2 Predictors of the latent parallel model

Our study identified predictive factors associated with various latent profile categories, including sex, per capita monthly family income, adherence to physical activity, and alcohol consumption. First, our study revealed that girls are more likely to experience severe negative emotional states compared to boys, consistent with previous research findings (Tao et al., 2024; Liu et al., 2019; Zhang X. et al., 2024). Secondly, college students with lower per capita monthly family income are likely to experience more severe negative emotional states. With the accelerating pace of life, college students from economically disadvantaged families face not only academic pressures but also concerns about future career development and family financial responsibilities, making them more susceptible to anxiety or depression (Markoulakis et al., 2020). Thirdly, the results indicate that college students who have not exercised regularly in the past month may exhibit more severe negative emotional states. This may be because regular physical activity enhances emotional stability and overall emotional resilience, positively influencing college students’ emotional states (Mu et al., 2024). Fourthly, college students who have consumed alcohol in the past month are more likely to experience severe negative emotional states. Previous studies have shown a significant association between alcohol consumption and negative emotions among college students, with those who engage in heavy drinking not only harming their health but also experiencing anxiety and depression (Nourse et al., 2017). These findings suggest that female students, those with lower per capita monthly family income, those who lack regular exercise, and those with drinking alcohol habits should receive focused attention. Early identification and intervention for students likely to experience severe negative emotional states are crucial, and symptomatic treatment should be provided when necessary.

### 4.3 e-Health literacy

This study chose e-HL as an associated variable of negative emotion characteristics because previous research has shown that college students’ e-HL is closely related to anxiety and depression symptoms (Zhang S. et al., 2024; Lin et al., 2024). The more severe the anxiety and depression symptoms, the lower the e-HL level among college students. When college students experience anxiety or depression, their self-management behaviors become less regulated, affecting their psychological help-seeking behaviors and health behaviors, which in turn impacts their e-HL level. Notably, this relationship may be confounded or moderated by digital addiction—a prevalent issue among college students that frequently co-occurs with negative emotional symptoms (Zhang and Wang, 2024; Zhang J. et al., 2024). Recent evidence indicates that digital addiction is strongly associated with both depression/anxiety and impaired e-HL: individuals with digital addiction may exhibit compulsive online behaviors that reduce the quality of health information processing while exacerbating negative emotions through social comparison or information overload (Zhang and Wang, 2024). Conversely, negative emotional symptoms may increase the risk of digital addiction as a coping mechanism, creating a bidirectional cycle that indirectly impairs e-HL (Zhang J. et al., 2024). Our study revealed differences in e-HL across different latent profile categories, with individuals in the high negative emotions group having lower e-HL levels compared to those in the low/no negative emotions group. Research indicates that good e-HL skills are conducive to individuals purposefully acquiring health knowledge and improving health behaviors. The application competency dimension represents an individual’s ability to obtain health information online, while the judgment and decision-making ability dimension reflects an individual’s capacity to discern the authenticity of information based on sufficient health knowledge and skills. In the era of rapid information technology and internet development, the threshold for obtaining health knowledge from mobile phones and computers has become more accessible with the widespread use of the internet. Individuals with high e-HL are more likely to adopt healthy behaviors and make greater use of online health resources. When individuals experience negative emotions, they are more likely to seek scientifically correct methods to alleviate their negative emotions, thereby preventing or mitigating depressive symptoms to some extent (Shudayfat et al., 2023). However, the failure to account for digital addiction in the current study means we cannot rule out the possibility that the observed negative correlation between negative emotional symptoms and e-HL is partially driven by this confounding factor. For example, the high negative emotional symptom group may have higher rates of digital addiction, which independently reduces their e-HL, leading to an overestimation of the direct effect of negative emotions. Therefore, in the process of conducting mental health interventions for college students, it is essential to further strengthen students’ personal self-management, enhance their self-efficacy, encourage them to change unhealthy behaviors, and consolidate personal health-promoting behaviors to better improve their e-HL level. From a theoretical perspective, exploring the significant differences in e-HL among different subgroups helps deepen our understanding of the influencing factors related to negative emotions. Clinically, direct treatment methods focusing solely on negative emotions are not very effective. Our study suggests that improving college students’ e-HL levels may enhance the effectiveness of negative emotion treatments, providing a reliable direction for the clinical treatment of negative emotions.

### 4.4 Public health implications

Based on the study findings, we put forward the following evidence-based and targeted intervention strategies for college mental health management to address the identified high-risk groups and key influencing factors:

First and foremost, implement targeted stratified mental health screening: Prioritize high-risk subgroups (females, low-income students, inactive individuals, and drinkers) for bi-annual screening using GAD-7 and PHQ-9. For students categorized into the high negative emotional symptoms group, conduct quarterly re-screening and initiate a 72-h response mechanism—matching positive cases with a dedicated mental health counselor for a preliminary assessment and personalized support plan within 3 working days. For the low/no negative emotional symptoms group, deliver annual universal mental health checks combined with preventive education to maintain psychological resilience.

Secondly, carry out lifestyle-oriented intervention programs: For the high negative emotional symptoms group: Design a 12-week structured physical activity program (3 sessions/week, 40 min/session) combining aerobic exercise and mindfulness training, led by certified fitness instructors and psychological counselors. Simultaneously, implement a peer support program for alcohol reduction—pairing students with drinking habits with non-drinking peers for weekly check-ins, and offering a “low-alcohol social skills” workshop to address social drinking triggers. For the low/no negative emotional symptoms group: Promote campus-wide fitness challenges with incentives and launch “healthy socialization” campaigns to reinforce positive lifestyle norms.

Thirdly, strengthen e-HL enhancement initiatives: For the high negative emotional symptoms group: Deliver an 8-week integrated workshop (1 session/week, 90 min/session) combining e-HL and digital addiction prevention. Core modules include: identifying credible mental health resources (e.g., national psychological service platforms, academic databases), critical evaluation of online health misinformation, and practical skills for controlled online use (e.g., app-based screen time management, setting information-seeking time limits). Provide 1-on-1 coaching (3 sessions total) for students with low e-HL scores (<25 points) to address individual gaps. For the low/no negative emotional symptoms group: Develop a self-paced online course (6 modules, 20 min/module) on e-HL basics (e.g., efficient health information search strategies, utilizing online counseling resources) and issue a “digital health passport” for course completion, encouraging proactive use of online mental health tools.

Fourthly, provide targeted support for students from low-income families: Establish a “financial-psychological integration support package” for low-income students in the high negative emotional symptoms group: Combine need-based scholarships (with simplified application procedures and 7-day feedback) with monthly one-on-one psychological counseling (focused on economic stress coping and future planning) and peer mentorship programs (pairing with senior students who have overcome similar challenges). For low-income students in the low/no negative emotional symptoms group: Offer part-time work-study opportunities related to mental health promotion (e.g., campus mental health ambassadors) to enhance self-efficacy, and provide free access to e-HL workshops and fitness program resources.

Fifthly, integrate digital addiction intervention into profile-specific mental health care: For the high negative emotional symptoms group: Embed digital addiction screening into routine mental health evaluations. For students with moderate-to-high digital addiction scores, add 4 specialized sessions on digital behavior regulation to their personalized support plan. For the low/no negative emotional symptoms group: Launch campus-wide “digital well-being” campaigns to prevent the onset of problematic digital use and maintain healthy online behaviors.”

### 4.5 Limitations and future directions

This study has several limitations: (1) Self-report measures may introduce bias, and future studies should combine clinical interviews for validation; (2) The cross-sectional design cannot establish causality, and longitudinal studies are needed to explore bidirectional relationships between negative emotions and e-HL; (3) Convenience sampling with uneven gender and grade distribution limits generalizability, so future studies should use stratified sampling to ensure representativeness; (4) Key variables such as social support and academic stress were not included in the study, and future research should incorporate these to explore more comprehensive mechanisms. (5) Digital addiction was not measured, which may confound the relationship between negative emotional symptoms and e-HL. Future studies should include digital addiction scales to disentangle these relationships.

## 5 Conclusion

This study identified two latent profiles of negative emotions among Chinese college students using LPA, with sex, family income, physical activity, and alcohol consumption as key predictors. e-HL was negatively associated with negative emotional symptom severity. The findings emphasize the need for early screening of high-risk groups and targeted interventions to improve mental health outcomes, providing a scientific basis for college mental health management.

## Data Availability

In accordance with the stipulated data use agreement, applicants are obligated to sign a data use agreement and specify the intended use of the study. Following theacquisition of approval from the Southern Medical University Bioethics Committee, applicants can formally request deidentified data and analysis scripts from the corresponding author (YQ).

## Footnotes

1
https://www.wjx.cn/

2
https://www.ibm.com/spss
